# Quality of life assessment in diffuse large B-cell lymphoma (DLBCL) in REFLECT: a prospective, non-interventional, multicenter, German study, assessing Sandoz rituximab in combination with CHOP

**DOI:** 10.1007/s00277-024-05850-5

**Published:** 2024-06-20

**Authors:** Boris Kubuschok, Burkhard Otremba, Manfred Welslau, Julian Topaly, Thomas Wolff, Georg Lenz, Michael Grau, Larissa Bittencourt da Silva, Ines Brückmann, Tobias Foierl

**Affiliations:** 1 Department of Internal Medicine II (Hematology/Oncology) and Comprehensive Cancer Center Augsburg, CCC Alliance WERA and Bavarian Cancer Research Center (BZKF), Universitätsklinikum Augsburg, Augsburg, Germany; 2 Onkologische Praxis Oldenburg, Oldenburg, Germany; 3 Comprehensive Cancer Center Mainfranken, Onkologie Aschaffenburg, Germany and Comprehensive Cancer Center Mainfranken, CCC Alliance WERA and BZKF, Würzburg, Germany; 4 MVZ Klinikum Osnabrück GmbH, Osnabrück, Germany; 5 OncoResearch Lerchenfeld, Hamburg, Germany; 6 Department of Medicine A for Hematology, Oncology and Pneumology, Universitätsklinikum Münster, Münster, Germany; 7 Sandoz, Holzkirchen, Germany

**Keywords:** Biosimilar, Rituximab, Quality of life, Real world, R-CHOP, DLBCL

## Abstract

Health-related quality of life (HRQoL) data are important indicators of health status in patients with lymphoma. The objective of this analysis was to assess the impact of treatment with Sandoz rituximab plus cyclophosphamide, doxorubicin, vincristine, and prednisone (R-CHOP) on HRQoL in treatment-naïve adult patients with diffuse large B-cell lymphoma (DLBCL) included in the prospective, real-world REFLECT study. REFLECT is the first prospective study to assess HRQoL in patients with DLBCL treated with a rituximab biosimilar. HRQoL was assessed via the patient-reported European Organization for Research and Treatment of Cancer Core Quality of Life questionnaire at baseline, mid-treatment (month 3), end of treatment (month 6), and follow-up (months 9 and 12). Subgroup analyses were performed to evaluate the influence of baseline characteristics on HRQoL, and associations between baseline HRQoL and treatment response. HRQoL was assessed in 169 patients. Mean global health status score remained stable from baseline (54.8) to mid-treatment (month 3; 54.7), before steadily improving through to end of treatment (month 6; 61.4), and follow-up month 9 (64.9) and month 12 (68.8). Similar trends were observed across most functional and symptom subscales. Higher cognitive, physical, or role functioning, and less appetite loss, diarrhea, fatigue, or pain at baseline, were all associated with an improved likelihood of reaching a complete versus partial response at the end of treatment. Overall, these findings confirm the HRQoL benefits of R-CHOP therapy in treatment-naïve adult patients with DLBCL, and suggest that baseline HRQoL may be predictive of treatment response.

**Supplementary Information:**

The online version contains supplementary material available at 10.1007/s00277-024-05850-5.

## Introduction

Diffuse large B-cell lymphoma (DLBCL) is the most frequent form of non-Hodgkin lymphoma among adults, accounting for approximately 30% of cases [1, 2]. DLBCL has an aggressive disease course, and without treatment, median survival can be less than one year [2, 3].

The chemotherapy regimen cyclophosphamide, doxorubicin, vincristine, and prednisone (CHOP) has been used in the treatment of lymphoma for over 40 years [4, 5]. Rituximab in combination with CHOP (R-CHOP) is an established standard of care in several countries for patients with newly diagnosed DLBCL [5], and can be curative in up to 60% of patients with de novo DLBCL [2].

Sandoz rituximab (SDZ-RTX; Rixathon^®^) received regulatory approval as a rituximab biosimilar in the European Union in 2017 [6]. Based on the totality of evidence for biosimilarity, SDZ-RTX is approved for use in the same indications as reference rituximab (MabThera^®^, Roche Pharmaceuticals), and is therefore approved for the treatment of adult patients with CD20-positive DLBCL in combination with CHOP [7]. The prospective, multicenter, open-label, non-interventional REFLECT study was designed to assess the efficacy and safety of SDZ-RTX plus CHOP chemotherapy in treatment-naïve patients with CD20-positive DLBCL [8]. REFLECT was the first prospective study of SDZ-RTX in patients with DLBCL. REFLECT reconfirmed the safety and effectiveness of SDZ-RTX in combination with CHOP as a first-line therapy in patients with DLBCL treated in a real-world setting [8].

Health-related quality of life (HRQoL) and other patient-reported outcomes are important indicators of patients’ health status, and correlate with established disease-specific outcome measures [9, 10]. Evidence shows that quality of life is a prognostic indicator for survival in patients with DLBCL, including those receiving R-CHOP [10–12]. DLBCL is a disease with high symptom burden, especially when compared to other cancers, and is therefore often accompanied by a deterioration in various domains of HRQoL [13]. Patients receiving chemotherapy may also report high levels of psychological distress and lower overall HRQoL compared with normative populations [14–16]. Studies in DLBCL survivors have shown greater HRQoL impairment in younger versus older patients, and in female versus male patients [13, 16]. Various instruments are available to assess HRQoL, including cancer-specific tools such as the European Organization for Research and Treatment of Cancer Core Quality of Life questionnaire (EORTC QLQ-C30) and Functional Assessment of Cancer Therapy-General, as well as generic measures such as the EuroQol-5 Dimensions and the 36-Item Short Form Health Survey [17].

The REFLECT study assessed the real-world impact of R-CHOP therapy on HRQoL in patients with DLBCL, using the patient-reported EORTC QLQ-C30. The influence of baseline characteristics on HRQoL trajectories during treatment, and any associations between baseline HRQoL and treatment response, was also evaluated.

## Methods

### Study design

The design and methodology of REFLECT have been published previously [8]. Briefly, REFLECT was a real-world, prospective, observational, multicenter, open-label, single-arm, non-interventional study in treatment-naïve adult patients with CD20-positive DLBCL, conducted across Germany. Patients received SDZ-RTX in combination with CHOP (R-CHOP), as per the treating physician’s discretion. Data were recorded from routine clinical practice, and no study-specific treatment regimens, assessments, or visit schedules were required.

### Patients

In accordance with the SDZ-RTX Summary of Product Characteristics (SmPC) [6], eligible patients were aged ≥ 18 years with a confirmed diagnosis of CD20-positive DLBCL, eligible for R-CHOP therapy based on physician’s discretion, and who had provided their written informed consent prior to entry into the study. Patients who had received prior therapy for DLBCL or who had any contraindications according to the SDZ-RTX SmPC [6] were excluded.

### Therapy

Patients received R-CHOP chemotherapy at visits 1 to 6 or 8, with the dosing schedule determined by the treating physician (R-CHOP14 infused once every 2 weeks or R-CHOP21 infused once every 3 weeks). Up to 8 cycles were administered at the discretion of the treating physician.

### HRQoL assessments

HRQoL was assessed as a secondary endpoint in REFLECT using the patient-reported EORTC QLQ-C30 at baseline and at month 3 (mid-treatment), month 6 (end of treatment), and months 9 and 12 (follow-up).

The EORTC QLQ-C30 is a standardized tool that incorporates a global health status/HRQoL scale, five functional scales (physical, role, emotional, cognitive, and social), three symptom scales (fatigue, nausea/vomiting, and pain), and six single-item measures (dyspnea, insomnia, appetite loss, constipation, diarrhea, and financial impact). All scores could range from 0 to 100, with rising scores on functional and global health status/HRQoL scales indicating improvement, and rising scores on symptom/single-item scales indicating worsening [18].

Subgroup analyses were performed to evaluate changes in HRQoL over time, based on the following patient baseline characteristics: age (< 65 vs. ≥ 65 years), sex (male vs. female), disease stage (Ann Arbor Stage I/II vs. Stage III/IV), International Prognostic Index (IPI) score (0–2 vs. 3–5), use of key concomitant medications (use vs. no use; key concomitant medications defined as any of the following: corticosteroids for systemic use, analgesics, antiemetics and antinauseants, antibacterial for systemic use and antihistamines for systemic use), presence of any medical event in patient history (present vs. absent), and presence of any serious medical event in patient history (present vs. absent; serious medical events defined as any of the following: cardiac failure, left ventricular failure, renal failure, hepatic cirrhosis, asthma, chronic obstructive pulmonary disease, pulmonary embolism, pulmonary fibrosis, Parkinson’s disease, autoimmune thyroiditis, rheumatoid arthritis, or psoriasis).

The association between global health status at baseline and the chance of reaching a complete response (CR) versus a partial response (PR), based on best overall response (BOR) during the study or response at the end of treatment, were also evaluated.

### Data analysis

Analyses were carried out on the full analysis set (FAS), which included all patients who received at least one dose of R-CHOP. Treatment response was recorded in each participating study center. The time of enrollment into the study was defined as the point of signing informed consent. All data analyses were performed by the sponsor (Sandoz).

Continuous variables are summarized by number of patients, mean, standard deviation, minimum, and maximum; for selected parameters, 25th and 75th percentiles are also presented. Categorical variables are summarized by number of patients and percentages.

In the subgroup analyses, for evaluation of outcomes between two groups, a t-test was performed, and *p*-values were reported. Two t-tests were carried out, Pooled and Satterthwaite, under variance equal and unequal. Under equality of variances, if *p* > 0.05, then the *p*-value corresponding to the Pooled test is reported; if *p* ≤ 0.05, then the *p*-value corresponding to the Satterthwaite test is reported.

This study was designed, implemented, and reported in accordance with the Guidelines for Good Pharmacoepidemiology Practices of the International Society for Pharmacoepidemiology and the Strengthening the Reporting of Observational Studies in Epidemiology guidelines [19].

## Results

### Patient characteristics and treatment

The REFLECT study enrolled 184 treatment-naïve adult patients with CD20-positive DLBCL. The FAS consisted of 169 patients who received at least one dose of R-CHOP.

Demographics and baseline characteristics of patients in the FAS are shown in Table 1. The median age (range) was 70 (24–94) years, and there were slightly more females than males (52.1% vs. 47.9%). Most patients (80.5%) had an Eastern Cooperative Oncology Group performance status of 0 or 1 at baseline. In total, 19.5% (*n* = 33/169) and 24.3% (*n* = 41/169) of patients had an Ann Arbor disease stage of III/IV, respectively. Overall, 75.1% of patients received R-CHOP14 and 24.9% received R-CHOP21.

**Table 1:** Patient demographics and baseline characteristics (FAS population)

Characteristic	Patients (*N* = 169)
Age at baseline, years	
Mean (SD)	67.3 (13.4)
Q1–Q3	58.0–78.0
Min–Max	24–94
Age group, n (%), years	
< 60	47 (27.8)
≥ 60	122 (72.2)
Sex, n (%)	
Female	88 (52.1)
Male	81 (47.9)
ECOG performance status at baseline, n (%)	
0	58 (34.3)
1	78 (46.2)
2	8 (4.7)
3	3 (1.8)
Not available	22 (13.0)
IPI score, n (%)	
0	11 (6.5)
1	38 (22.5)
2	36 (21.3)
3	37 (21.9)
4	17 (10.1)
5	2 (1.2)
Missing	28 (16.6)
Ann Arbor staging	
I	45 (26.6)
II1	35 (20.7)
II2	13 (7.7)
III	33 (19.5)
IV	41 (24.3)
Not available	2 (1.2)

Age was calculated from date of screening and date of birth

ECOG, Eastern Cooperative Oncology Group; FAS, full analysis set; IPI, International Prognostic Index; SD, standard deviation

### HRQoL

**Fig. 1:**
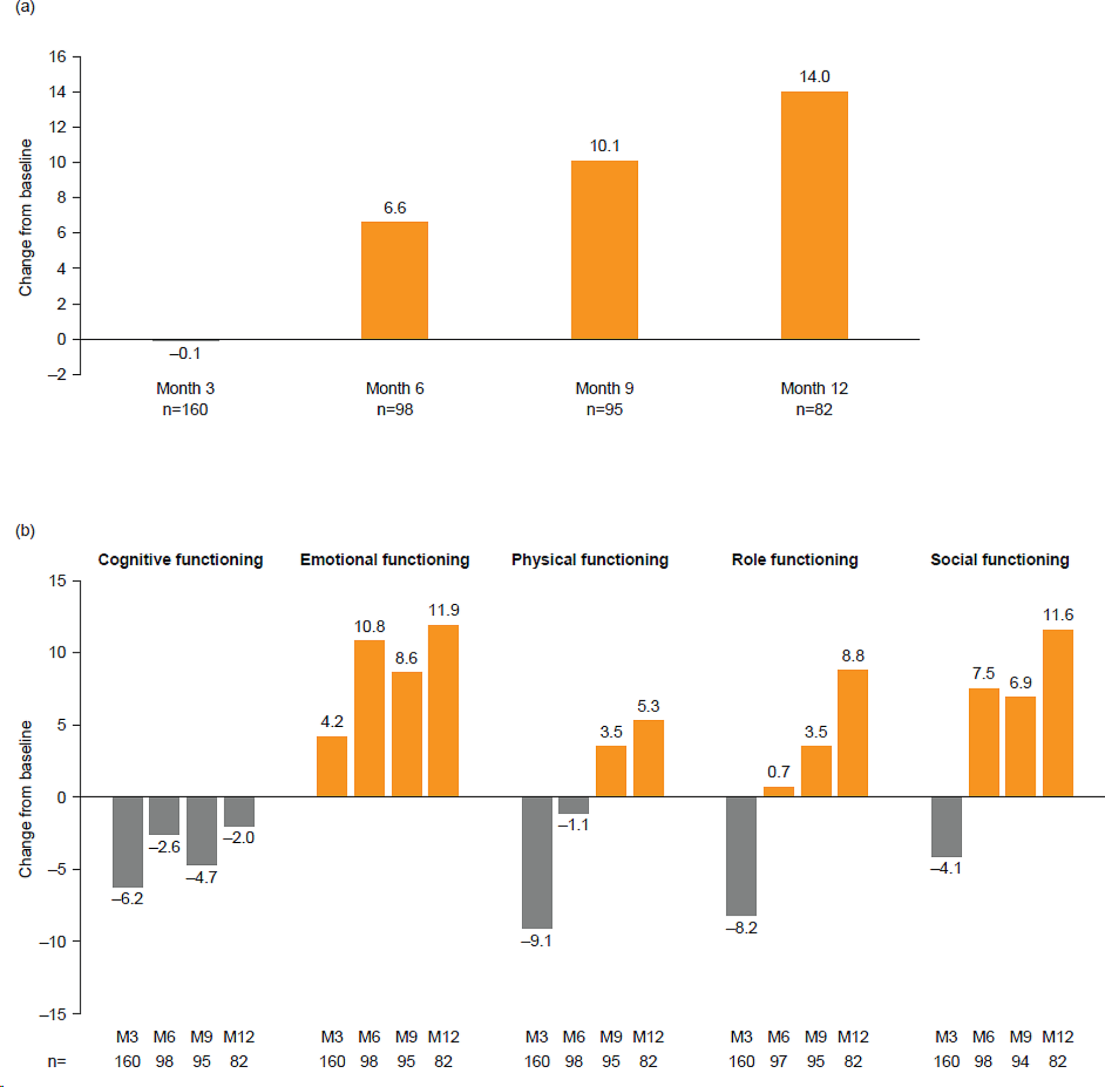
EORTC QLQ-C30 (**a**) global health status and (**b**) functional subscales: Mean change from baseline to months 3, 6, 9, and 12. Mean (SD) absolute values are shown in Supplementary Table 1. Month 3, mid-treatment; month 6, end of treatment; months 9 and 12, follow-up. At baseline: *n* = 165 for global health status, and cognitive, emotional, and physical functioning; *n* = 164 for role and social functioning. Decrease from baseline indicates a worsening in global health status/HRQoL or functioning; increase from baseline indicates an improvement in global health status/HRQoL or functioning. EORTC QLQ-C30, European Organization for Research and Treatment of Cancer Core Quality of Life questionnaire; HRQoL, health-related quality of life; M, month; SD, standard deviation

At baseline, the mean EORTC QLQ-C30 global health status score was 54.8 (*n* = 165). Mean global scores remained stable from baseline to mid-treatment (month 3 [*n* = 160]), before steadily improving through to end of treatment (month 6 [*n* = 98]) and follow-up (month 9 [*n* = 95] and month 12 [*n* = 82]), with an overall improvement of 14.0 points from baseline to month 12 (Fig. 1a).

Scores for the individual scales of physical functioning, role functioning, and social functioning worsened from baseline to mid-treatment (month 3), before improving to above baseline levels by month 12 (Fig. 1b). Mean scores for cognitive functioning also worsened from baseline to mid-treatment (month 3), before improving to near baseline levels at month 12. Mean scores for emotional functioning showed an early increase at mid-treatment (month 3), with continued improvement at the end of treatment (month 6) to month 12 (Fig. 1b; Supplementary Table 1).

**Fig. 2:**
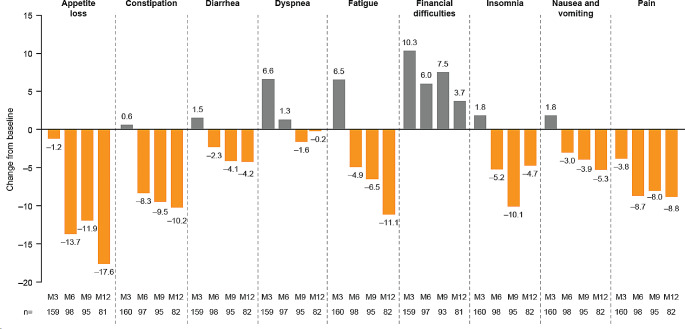
EORTC QLQ-C30 symptom scales: Mean change from baseline to months 3, 6, 9, and 12. Mean (SD) absolute values are shown in Supplementary Table 2. Month 3, mid-treatment; month 6, end of treatment; months 9 and 12, follow-up. At baseline: *n* = 165 for all domains apart from insomnia (*n* = 164). Decrease from baseline indicates symptom improvement; increase from baseline indicates symptom worsening. EORTC QLQ-C30, European Organization for Research and Treatment of Cancer Core Quality of Life questionnaire; M, month; SD, standard deviation

Mean symptom scale scores for appetite loss, constipation, diarrhea, fatigue, insomnia, and nausea and vomiting remained the same or worsened slightly at mid-treatment (month 3), and subsequently improved beyond baseline levels by month 12 (Fig. 2; Supplementary Table 2). For dyspnea, scores worsened from baseline to mid-treatment (month 3), before returning to baseline levels by month 12. Mean scores for pain improved during treatment and subsequent follow-up to month 12. Conversely, mean scores for financial difficulties worsened during treatment and follow-up (Fig. 2; Supplementary Table 2).

### Subgroup analyses – impact of baseline characteristics on HRQoL over time

#### Impact of age

**Fig. 3:**
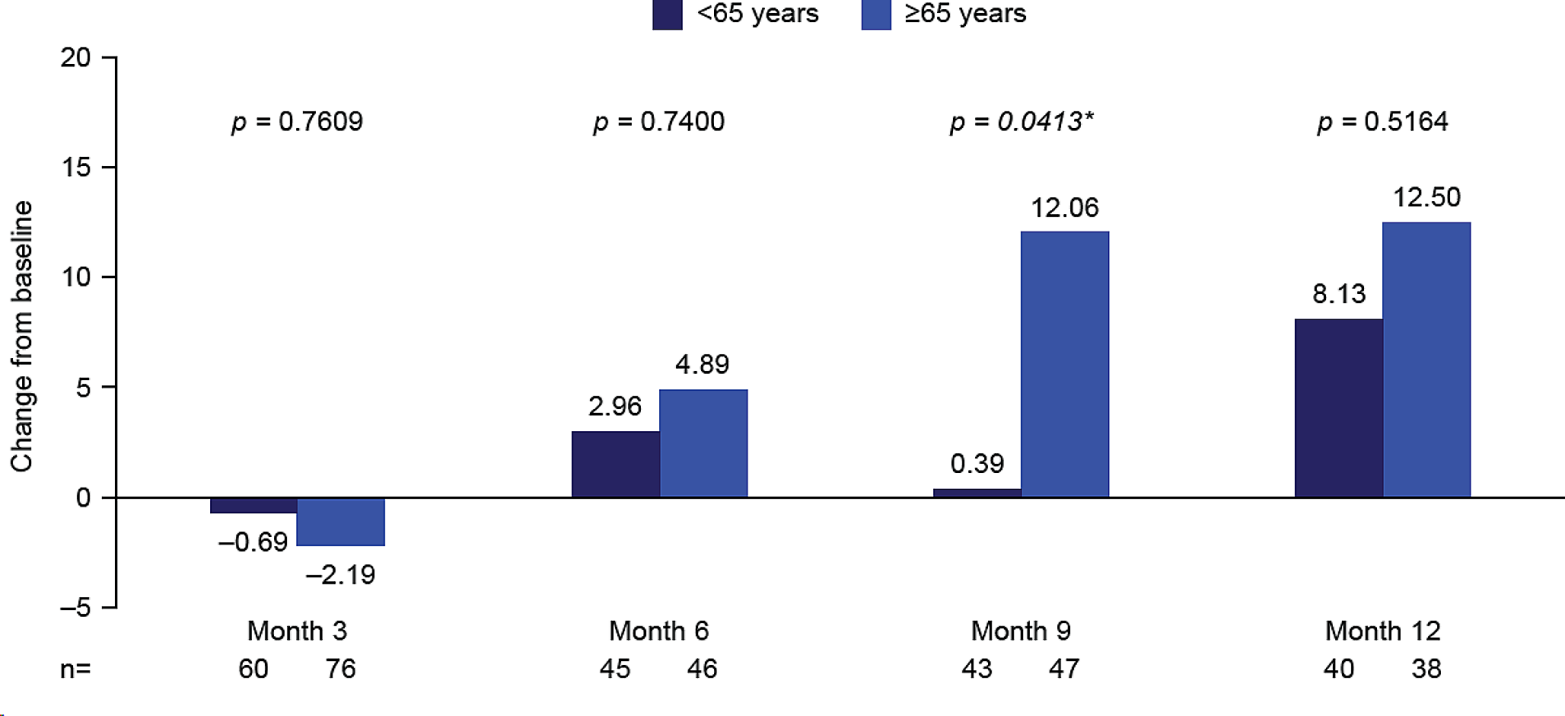
EORTC QLQ-C30 global health status stratified by age at baseline: Mean change from baseline to months 3, 6, 9, and 12. *p-value significant (t-test). Month 3, mid-treatment; month 6, end of treatment; months 9 and 12, follow-up. At baseline: *n* = 64 < 65 years, *n* = 101 ≥ 65 years. Decrease from baseline indicates a worsening in global health status/HRQoL; increase from baseline indicates an improvement in global health status/HRQoL. EORTC QLQ-C30, European Organization for Research and Treatment of Cancer Core Quality of Life questionnaire; HRQoL, health-related quality of life

Mean change from baseline in global health status score was significantly greater in older versus younger patients at follow-up month 9 (≥ 65 years [*n* = 47]: 12.06 vs. < 65 years [*n* = 43]: 0.39, *p* = 0.0413), but no statistically significant between-group differences were observed at any other time points (Fig. 3; Supplementary Table 3).

There was no clear trend for an impact of age across functional and symptom scales (Supplementary Table 3).

#### Impact of sex

**Fig. 4:**
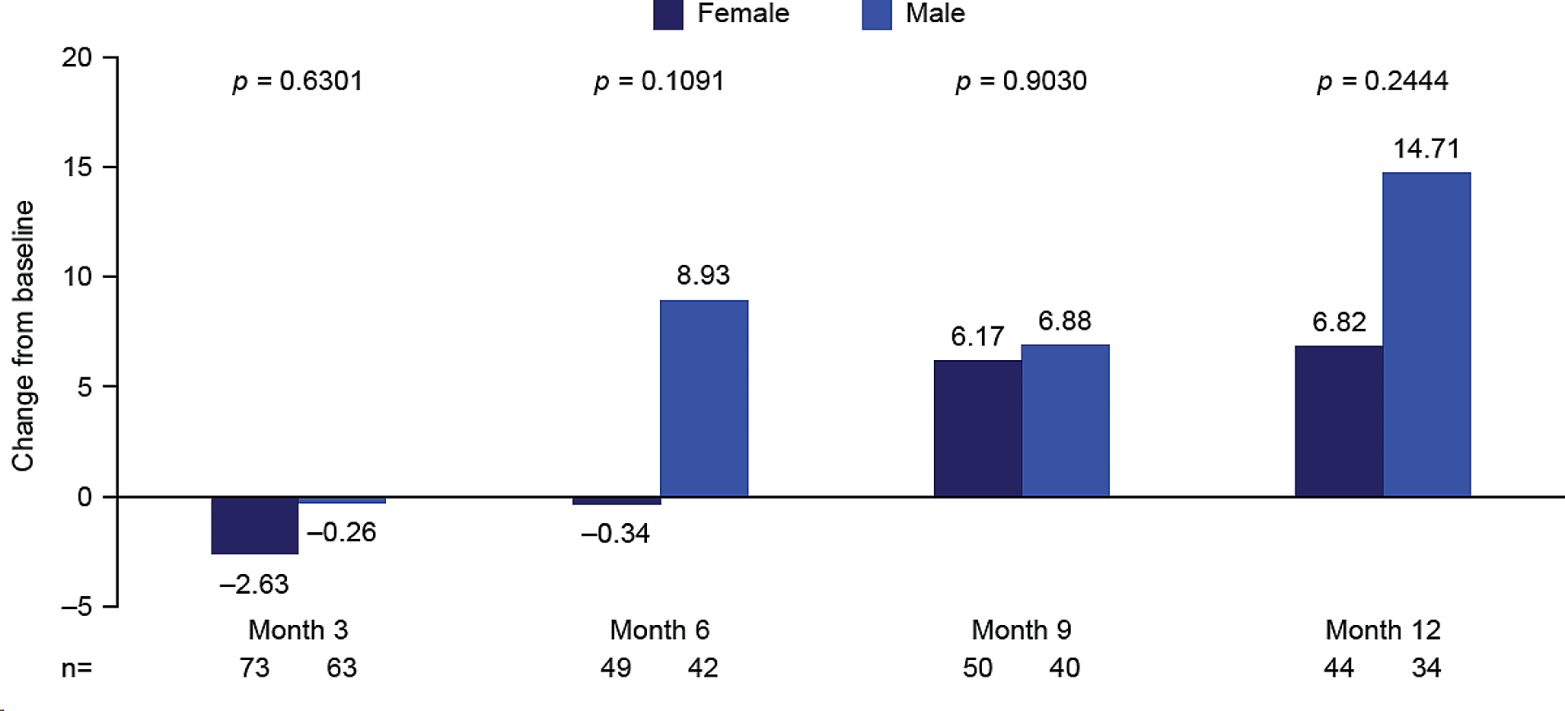
EORTC QLQ-C30 global health status stratified by sex: Mean change from baseline to months 3, 6, 9, and 12. Month 3, mid-treatment; month 6, end of treatment; months 9 and 12, follow-up. At baseline: *n* = 86 females, *n* = 79 males. Decrease from baseline indicates a worsening in global health status/HRQoL; increase from baseline indicates an improvement in global health status/HRQoL. EORTC QLQ-C30, European Organization for Research and Treatment of Cancer Core Quality of Life questionnaire; HRQoL, health-related quality of life

Mean changes from baseline in global health status score were not significantly different between male and female patients at any time point (Fig. 4; Supplementary Table 4).

A trend towards reduced improvement from baseline in functional subscales was evident for female versus male patients at all time points. Differences reached statistical significance for cognitive, emotional, physical, and role functioning (*p* = 0.0029, *p* = 0.0110, *p* = 0.0122 and *p* = 0.0024, respectively) at end of treatment (month 6), and cognitive and emotional functioning at follow-up month 12 (*p* = 0.0188 and *p* = 0.0483, respectively; Supplementary Table 4).

Numerically smaller improvements in fatigue, insomnia, and pain scores from baseline to end of treatment (month 6) and follow-up (months 9 and 12) were observed in female versus male patients. However, a statistically significant between-group difference was only reached for fatigue and dyspnea at end of treatment (month 6; *p =* 0.0120 and *p* = 0.0365) and appetite loss at follow-up month 9 (*p* = 0.0400; Supplementary Table 4). There were no clear trends indicating an impact of sex on any other symptom scales.

#### Impact of disease stage

**Fig. 5:**
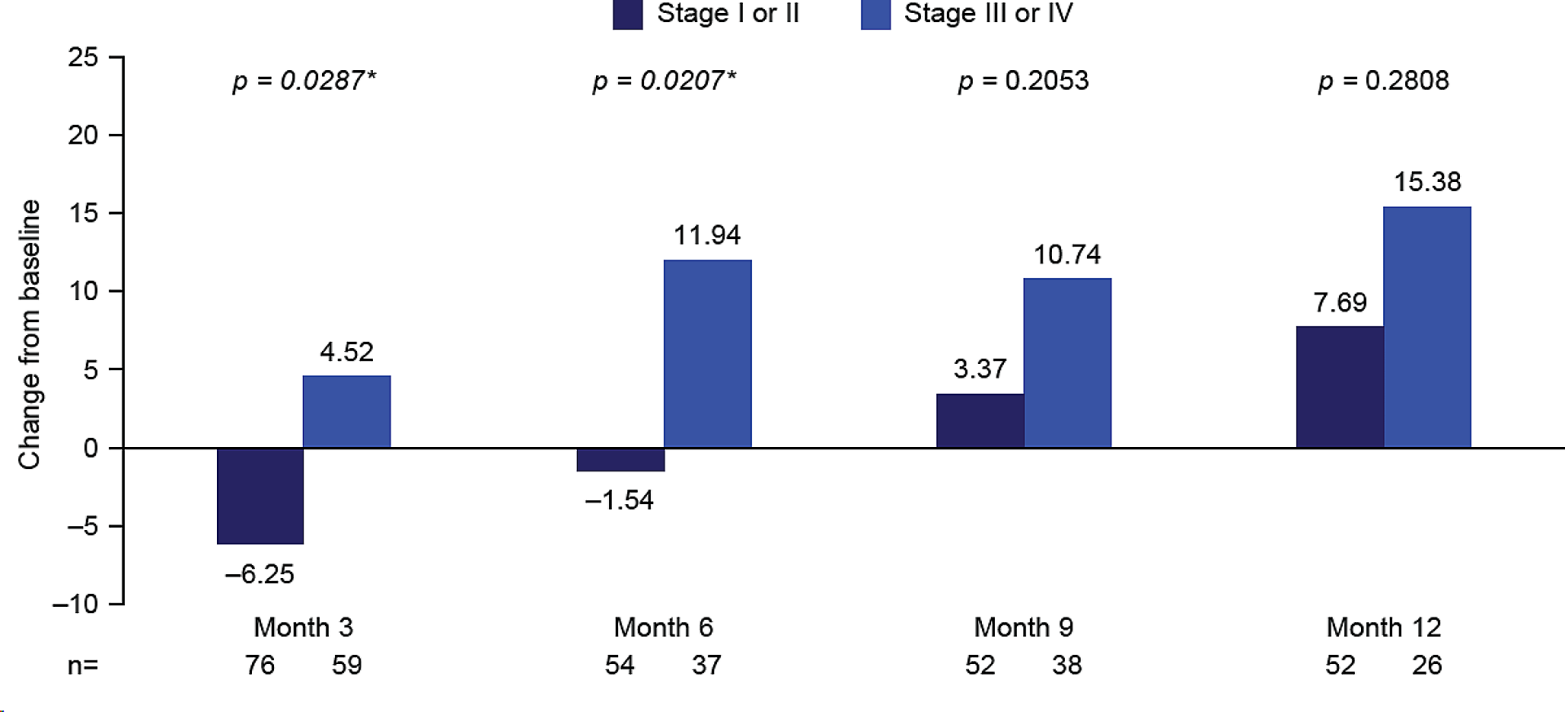
EORTC QLQ-C30 global health status stratified by disease stage at baseline: Mean change from baseline to months 3, 6, 9, and 12. *p-value significant (t-test). Month 3, mid-treatment; month 6, end of treatment; months 9 and 12, follow-up. At baseline: *n* = 90 Stage I/II, *n* = 74 Stage III/IV. Decrease from baseline indicates a worsening in global health status/HRQoL; increase from baseline indicates an improvement in global health status/HRQoL. EORTC QLQ-C30, European Organization for Research and Treatment of Cancer Core Quality of Life questionnaire; HRQoL, health-related quality of life

Mean changes from baseline in global health status scores showed a significantly greater improvement in patients with Ann Arbor Stage III/IV disease versus patients with Stage I/II disease at mid-treatment (month 3: mean change from baseline 4.52 vs. − 6.25; *p* = 0.0287), and at the end of treatment (month 6: mean change from baseline 11.94 vs. − 1.54; *p* = 0.0207). The same trend was observed during follow-up (months 9 and 12), but statistical significance was not reached (Fig. 5; Supplementary Table 5).

A similar pattern was observed in functional scale scores, but differences between the groups only reached significance at follow-up month 12 for physical functioning (*p* = 0.0424) and role functioning (*p* = 0.0175; Supplementary Table 5).

Across most symptom scales, patients with Stage I/II disease showed numerically less improvement from baseline to all time points versus patients with Stage III/IV disease (Supplementary Table 5).

#### Impact of IPI score

**Fig. 6:**
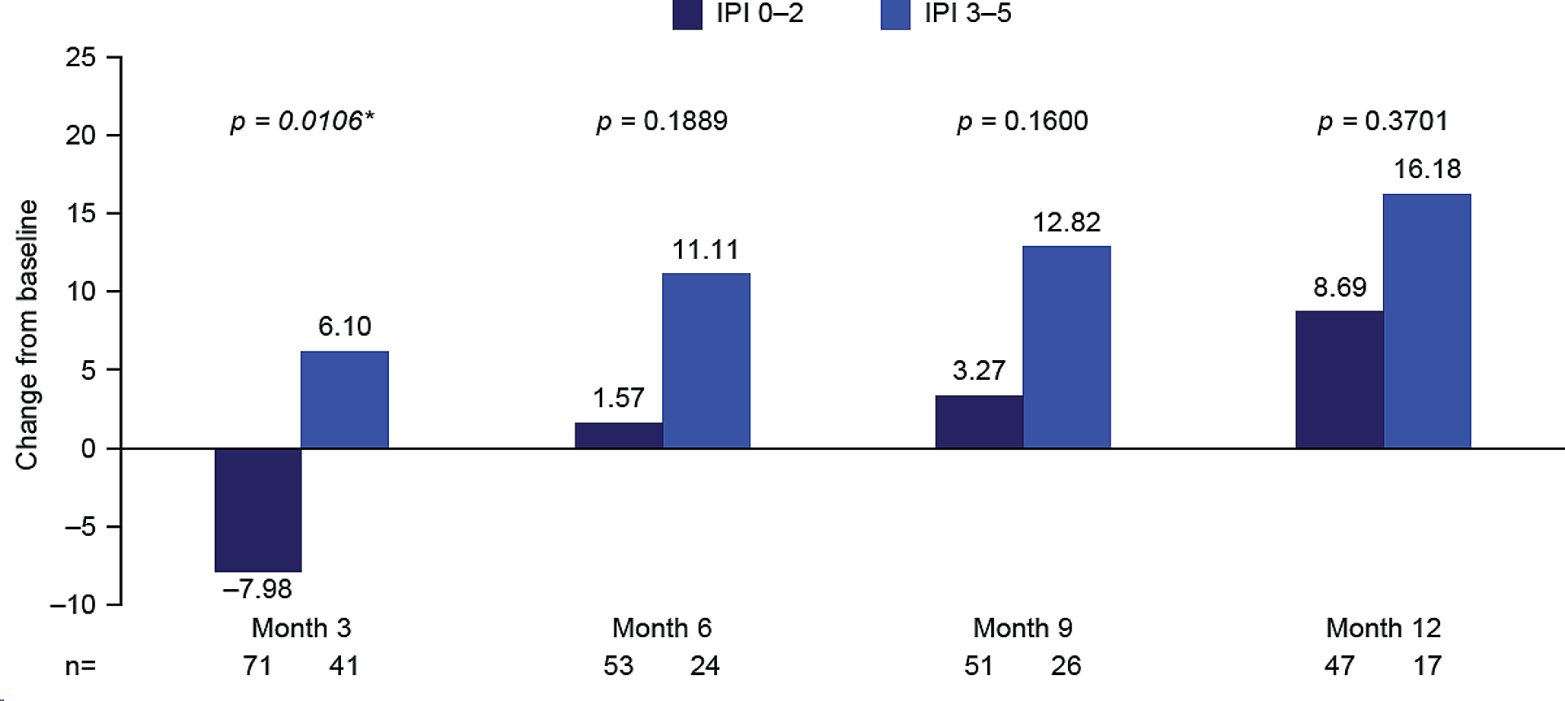
EORTC QLQ-C30 global health status stratified by IPI score at baseline: Mean change from baseline to months 3, 6, 9, and 12. *p-value significant (t-test). Month 3, mid-treatment; month 6, end of treatment; months 9 and 12, follow-up. At baseline: *n* = 83 IPI 0–2; *n* = 56 IPI 3–5. Decrease from baseline indicates a worsening in global health status/HRQoL; increase from baseline indicates an improvement in global health status/HRQoL. EORTC QLQ-C30, European Organization for Research and Treatment of Cancer Core Quality of Life questionnaire; HRQoL, health-related quality of life; IPI, International Prognostic Index

Mean changes from baseline in global health status score demonstrated a greater improvement in patients who had an IPI score of 3–5 at baseline compared with those who had an IPI score of 0–2, with significance observed at mid-treatment (month 3; 6.10 vs. − 7.98; *p* = 0.0106; Fig. 6; Supplementary Table 6).

At all time points, all symptom and single-item scores showed a numerically greater reduction in burden for patients who had an IPI score of 3–5 at baseline compared with those who had an IPI score of 0–2; appetite loss, fatigue, nausea and vomiting, and pain showed significance at ≥ 1 time point (Supplementary Table 6).

At all time points, functional scores showed a numerically greater improvement in patients who had an IPI score of 3–5 at baseline compared with those who had an IPI score of 0–2, with all but emotional and social functioning showing significance at ≥ 1 time point (*p* < 0.04). For role functioning, significance was observed at all time points (*p* < 0.02; Supplementary Table 6).

#### Impact of use of key concomitant medications

**Fig. 7:**
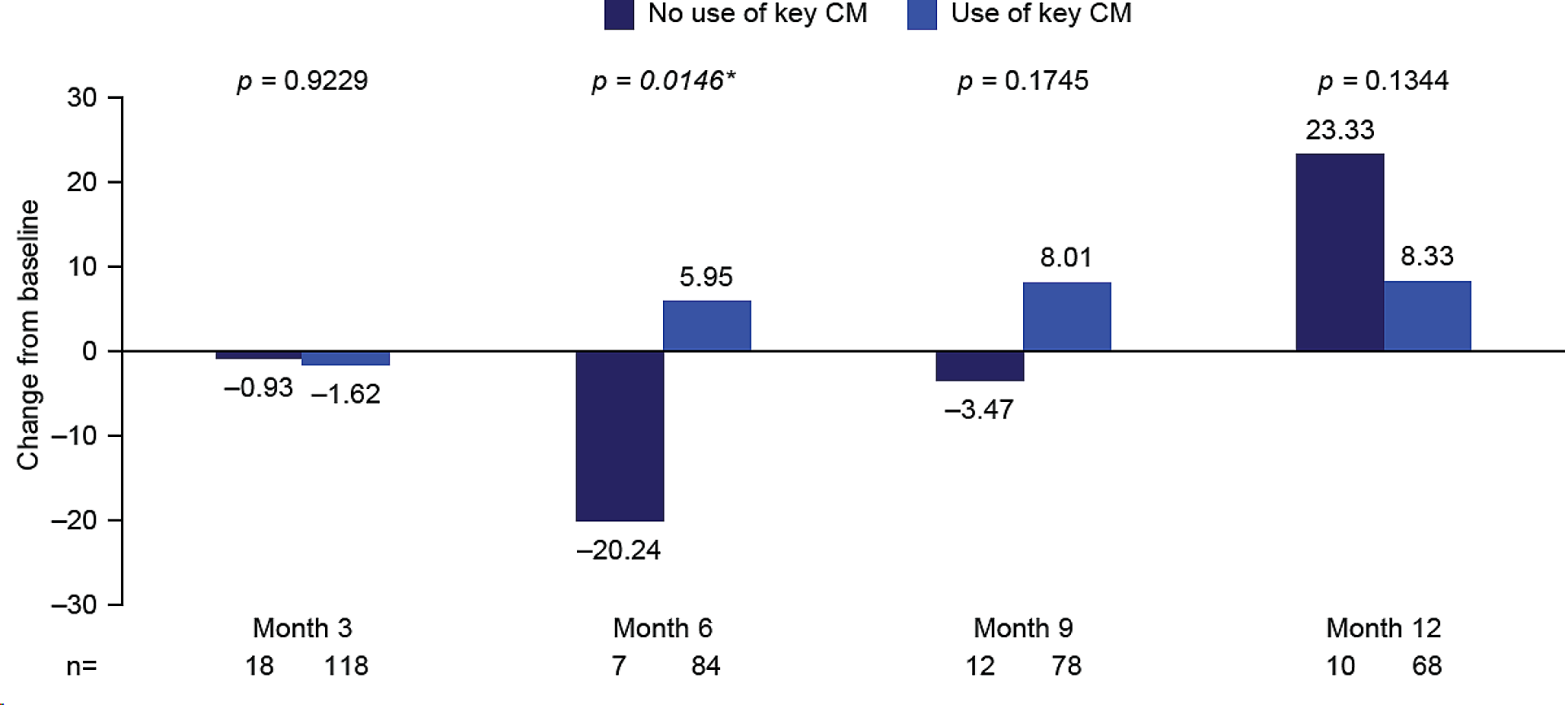
EORTC QLQ-C30 global health status stratified by use of any key concomitant medication at baseline: Mean change from baseline to months 3, 6, 9, and 12. *p-value significant (t-test). Month 3, mid-treatment; month 6, end of treatment; months 9 and 12, follow-up. At baseline: *n* = 23 for no use of key CM, *n* = 142 for use of key CM. Key CM defined as corticosteroids for systemic use, analgesics, antiemetics and antinauseants, antibacterial for systemic use, and antihistamines for systemic use. Decrease from baseline indicates a worsening in global health status/HRQoL; increase from baseline indicates an improvement in global health status/HRQoL. CM, concomitant medication; EORTC QLQ-C30, European Organization for Research and Treatment of Cancer Core Quality of Life questionnaire; HRQoL, health-related quality of life

Mean changes in global health status score showed a numerically greater improvement from baseline to end of treatment (month 6) and follow-up month 9 in patients who were receiving any of the key concomitant medications at baseline, compared with those who were not receiving any of these medications, with significance reached at the end of treatment (month 6: mean change from baseline 5.95 vs. − 20.24; *p* = 0.0146). However, the opposite trend was observed at follow-up month 12, with improvement in global health status score numerically greater in patients who were not receiving any of the key concomitant medications versus those who were (Fig. 7; Supplementary Table 7). These data must be interpreted with caution, as most patients (86%) were receiving at least one concomitant medication at baseline.

Inconsistent trends were also observed across the functional and symptom scales (Supplementary Table 7). For cognitive and social functioning, there was a trend for a greater improvement in patients who were not receiving key concomitant medication at baseline versus those who were, with significance reached for cognitive functioning at end of treatment (month 6; *p* = 0.0132) and social functioning at follow-up month 12 (*p* = 0.0107). For constipation, a numerically greater decrease in burden from baseline was observed across all time points for patients who were not receiving any of the key concomitant medications at baseline, versus those who were, with significance reached at mid-treatment (month 3) and follow-up month 12 (*p* = 0.0082 and *p* = 0.0425, respectively; Supplementary Table 7). However, for pain, a numerically greater increase in burden from baseline was observed at end of treatment (month 6) for patients who were not receiving any of the key concomitant medications at baseline, versus those who were (*p* = 0.027).

For financial difficulty, a numerically greater decrease in burden from baseline was observed across all time points for patients who were not receiving key concomitant medication versus those who were. This reached significance at mid-treatment (month 3) and follow-up month 9 (*p* = 0.0363 and *p* = 0.0185, respectively; Supplementary Table 7).

#### Impact of any medical event in patient history

**Fig. 8:**
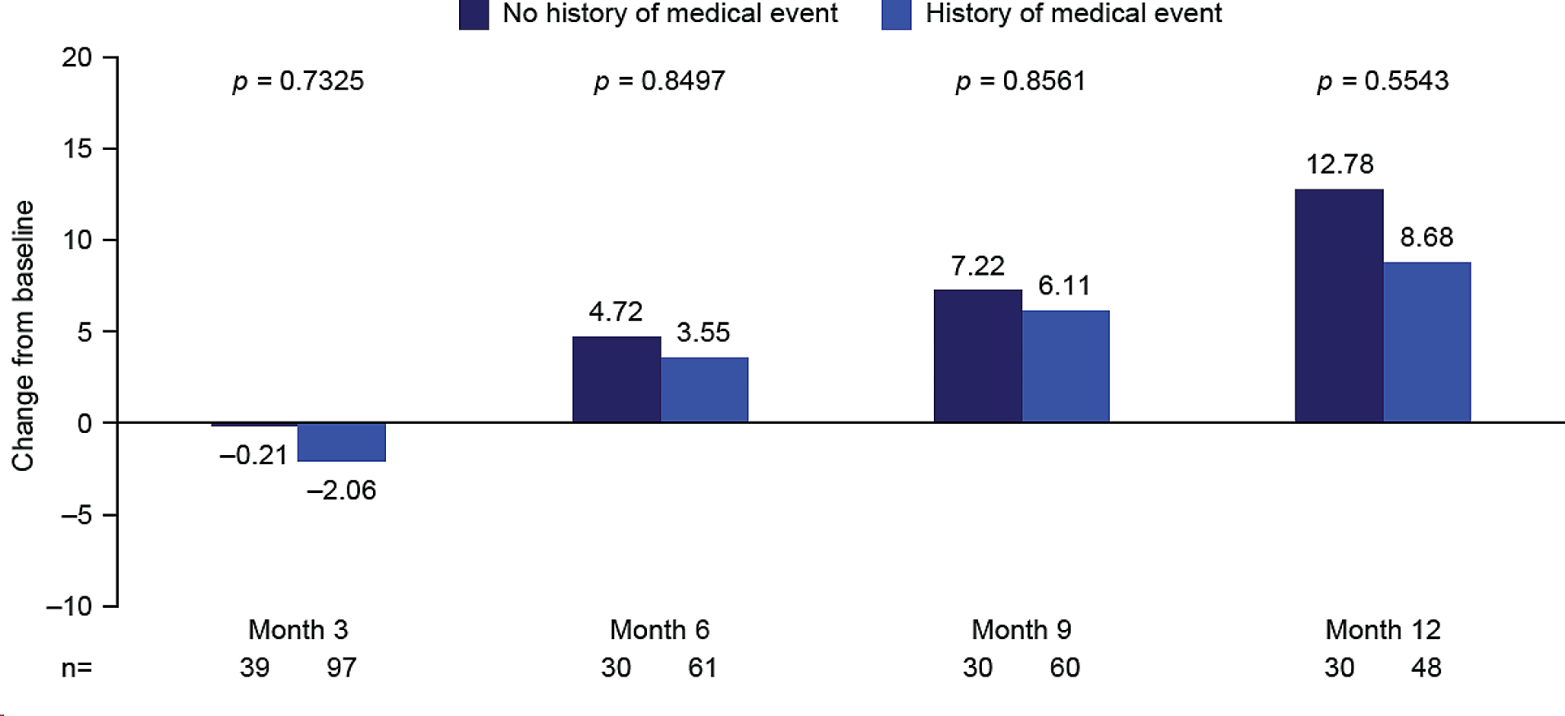
EORTC QLQ-C30 global health status stratified by the presence of any medical event in patient history: Mean change from baseline to months 3, 6, 9, and 12. Month 3, mid-treatment; month 6, end of treatment; months 9 and 12, follow-up. At baseline: *n* = 46 for no history of medical event, *n* = 119 for history of medical event. Decrease from baseline indicates a worsening in global health status/HRQoL; increase from baseline indicates an improvement in global health status/HRQoL. EORTC QLQ-C30, European Organization for Research and Treatment of Cancer Core Quality of Life questionnaire; HRQoL, health-related quality of life

A general trend of an improvement from baseline in mean global health status scores was observed over time for patients with and without a history of any medical event at baseline. Additionally, there was a numerically greater improvement from baseline in mean global health score for patients without a history of any medical event versus those with a history of any medical event. However, these values did not reach significance at any time point (Fig. 8; Supplementary Table 8).

Data for dyspnea showed a numerically greater decrease in burden for patients without a history of any medical event versus those with a history of any medical event, with significance reached at follow-up month 12 (*p* = 0.0024). No other clear trends were observed for any other symptom or functional scores (Supplementary Table 8).

#### Impact of any serious medical event in patient history

**Fig. 9:**
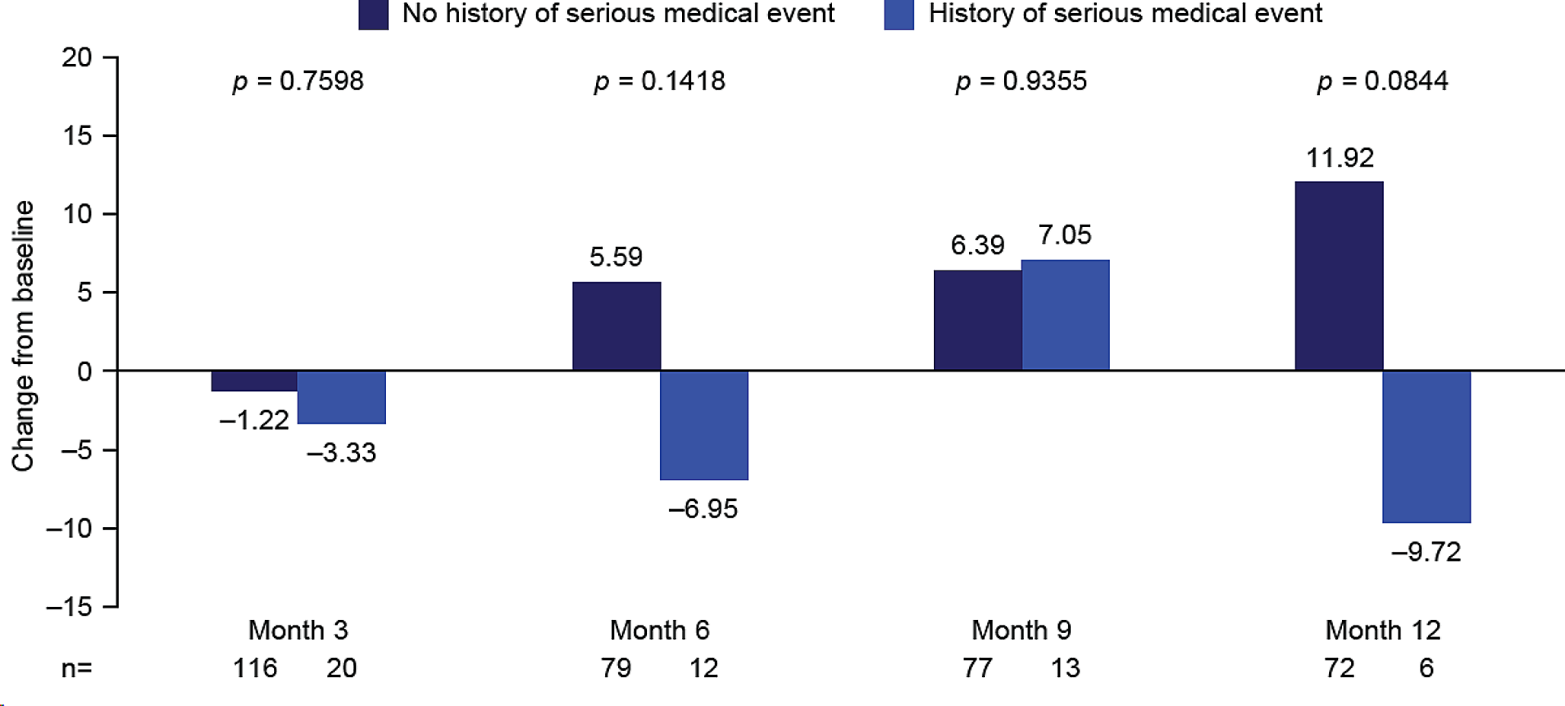
EORTC QLQ-C30 global health status stratified by the presence of any serious medical event in patient history: Mean change from baseline to months 3, 6, 9, and 12. Month 3, mid-treatment; month 6, end of treatment; months 9 and 12, follow-up. At baseline: *n* = 140 for no history of serious medical event, *n* = 25 for history of serious medical event. Serious medical event defined as any of the following: cardiac failure, left ventricular failure, renal failure, hepatic cirrhosis, asthma, chronic obstructive pulmonary disease, pulmonary embolism, pulmonary fibrosis, Parkinson’s disease, autoimmune thyroiditis, rheumatoid arthritis, or psoriasis. Decrease from baseline indicates a worsening in global health status/HRQoL; increase from baseline indicates an improvement in global health status/HRQoL. EORTC QLQ-C30, European Organization for Research and Treatment of Cancer Core Quality of Life questionnaire; HRQoL, health-related quality of life

Mean change from baseline in global health status score highlighted a numerical improvement at all time points from end of treatment (month 6) in patients without a history of any serious medical event at baseline. In patients with a history of any serious medical event, global health status score was only improved at follow-up month 9. There was no significant difference between the groups in change from baseline in global health status score at any time point (Fig. 9; Supplementary Table 9). However, these data must be interpreted with caution, as most patients (85%) did not have a history of any serious medical event.

Across most symptom scales, patients with a history of any serious medical event showed numerically less improvement from baseline to all time points versus patients without a history of any serious medical event. At follow-up month 9, significance was reached for nausea and vomiting (*p* = 0.0084); at follow-up month 12, significance was reached for pain and physical functioning scores (*p* = 0.0373 and *p* = 0.0476, respectively; Supplementary Table 9).

### Subgroup analyses – impact of baseline HRQoL on treatment response

There was no significant association between global health status at baseline and the chance of reaching a CR versus a PR, based on BOR during the study or response at the end of treatment (Supplementary Table 10).

Baseline scores on three functional scales were significantly predictive of reaching a CR versus a PR: patients with higher cognitive, physical, or role functioning at baseline were more likely to achieve a CR versus a PR using BOR (cognitive functioning, *p* = 0.0181; physical functioning, *p* = 0.0018; role functioning, *p* = 0.0038), or response at the end of treatment (cognitive functioning, *p* = 0.0091; physical functioning, *p* = 0.0103; role functioning, *p* = 0.0072; Supplementary Table 10).

Baseline scores on five symptom scales were significantly predictive of reaching a CR versus a PR: patients with less appetite loss, constipation, diarrhea, fatigue, or pain at baseline were more likely to achieve a CR versus a PR using BOR (appetite loss, *p* = 0.0259; constipation, *p* = 0.0364; diarrhea, *p* = 0.0412; fatigue, *p* = 0.0153; pain, *p* = 0.0098). Patients with less appetite loss, diarrhea, fatigue, or pain at baseline were more likely to achieve a CR versus a PR using response at the end of treatment (appetite loss, *p* = 0.0289; diarrhea, *p* = 0.0347; fatigue, *p* = 0.0033; pain, *p* = 0.0105; Supplementary Table 10).

## Discussion

The results from the REFLECT study demonstrate HRQoL benefit of biosimilar rituximab plus CHOP therapy in treatment-naïve patients with CD20-positive DLBCL, reflecting the real-world experience with reference rituximab plus CHOP. This is the first clinical or real-world study to report HRQoL data in patients with DLBCL receiving a rituximab biosimilar [20–25]. Additionally, this study reinforces the recent recommendations from oncological societies to obtain robust clinical data to ensure continued efficacy and safety of biosimilars [26, 27].

Most patients in the REFLECT study population were high functioning, but many had advanced disease. Patients’ HRQoL improved following R-CHOP therapy, with global health status scores remaining stable from baseline to mid-treatment (month 3), before steadily improving through to end of follow-up (month 12). An early improvement in emotional functioning was evident by mid-treatment, with continued improvement to follow-up month 12. Although physical, social and role functioning scores worsened initially, they subsequently improved to above baseline levels by month 12. Scores for symptom scales, including appetite loss, constipation, diarrhea, fatigue, insomnia, nausea, and vomiting remained the same or slightly worsened before improving over the follow-up period. Pain scores improved steadily over the 12-month study period; dyspnea scores worsened initially before returning to baseline levels. However, financial difficulties scores worsened across the study.

In the Phase III POLARIX trial comparing HRQoL in previously untreated patients with DLBCL receiving R-CHOP versus polatuzumab vedotin-R-CHOP, both regimens led to rapid and sustained improvements in HRQoL and symptoms [28]. Improvements in symptom scores were observed in most patients after Cycle 1, subsequently increasing until end of treatment. Overall, 81.3% of patients treated with R-CHOP achieved clinically meaningful improvement in symptom scores at any time point during the study, compared with 82.3% of patients receiving polatuzumab vedotin-R-CHOP [28]. Improvements in HRQoL from baseline to the end of treatment were similarly observed in REFLECT.

In REFLECT post hoc subgroup analyses, improvements in global health status scores were generally similar between older and younger patients, with no clear evidence of any impact of age across functional and symptom scales scores. This suggests that younger and older patients achieved similar HRQoL benefits from R-CHOP therapy in this study.

Although improvements in global health status scores were similar in female and male patients, the magnitude of improvement in functional subscale scores was smaller in females than males at all time points. Other studies have reported reduced HRQoL benefits following treatment in females versus males [16, 29]. In the REMoDL-B study of patients with DLBCL treated with R-CHOP and bortezomib, HRQoL was lower in females versus males, younger versus older patients, and those with Stage I versus Stage II–IV disease [29]. Among patients with DLBCL who received chemotherapy in the Real-Time Tailored Therapy study, mean EORTC QLQ‑C30 global health status and functional scale and symptom scores, notably the physical functioning and constipation subscales, were worse in females versus males at 1 year after diagnosis [16]. There are several possible reasons to explain the reduced HRQoL benefits in female versus male patients with cancer [30]. These include genetic differences between males and females [31], differences in the immune function [32], and differences in adverse drug reactions (e.g. more gastrointestinal symptoms and a higher risk for serious side effects have been observed in female versus male patients) [33]. Moreover, a potential impact of gender roles on subjective HRQoL has been observed in patients with coronary artery disease [34].

In analyses of the relationship between baseline HRQoL and treatment response, no significant association between global health status at baseline and the chance of reaching a CR versus a PR was observed in REFLECT. However, patients with higher cognitive, physical, or role functioning at baseline, and patients with less appetite loss, constipation, diarrhea, fatigue, or pain at baseline were more likely to achieve a CR versus a PR. This may be explained by the fact that better physical fitness is possibly correlated with fewer therapy-related side effects, stronger tumor immune responses, and better tumor control [35]. Although the underlying mechanisms are not completely elucidated, these effects can be partially assigned to systemic differences in host pathways, including metabolism, inflammation, and immune function, which promote a less tumor-promotive milieu [36]. Concerning cognitive function, data on cognitive function before start of therapy and its relationship to tumor control are too scarce to draw conclusions on the impact on reaching PR or CR [37].

These outcomes could also be the result of these patients generally exhibiting better health conditions before receiving chemotherapy. The impact of better health conditions prior to receiving this regimen could benefit the patient throughout treatment, and help mitigate any negative effects observed during chemotherapy, leading to an overall increased effectiveness. Patients with esophageal cancer who have undergone prehabilitation to improve strength and overall wellness demonstrated an improved rate of completion and overall tolerance to treatment with chemotherapy [38]. However, to confirm the utility of such prehabilitation approaches in DLBCL populations (such as included in the REFLECT study), further studies, especially prospective studies, are required.

Several studies have evaluated the effect of baseline HRQoL on survival following treatment. The GOYA Phase III trial demonstrated that pre-treatment EORTC QLQ-C30 physical functioning, global health status, and fatigue scores had high prognostic value for overall survival (OS) and progression-free survival in previously untreated patients with CD20-positive DLBCL receiving obinutuzumab/rituximab plus chemotherapy, even after adjustment for IPI score, cell of origin, B-cell lymphoma 2 mutation status, and total metabolic tumor volume [11]. A cohort study of R-CHOP therapy in patients with newly diagnosed DLBCL showed that pre-treatment HRQoL, assessed using the EORTC QLQ-C30, could independently predict OS [12]. Any potential association between pre-treatment HRQoL and overall survival was not assessed in this analysis of the REFLECT study.

Overall, in the RELFECT study, around 75% of patients were treated with biweekly CHOP14 compared with around 25% treated with CHOP21, with the choice of treatment at the discretion of the treating physician. This contrasted slightly with real-world data from the German Tumour Registry Lymphatic Neoplasms, which reported around 45% of patients with DLBCL were treated with CHOP14 and 55% were treated with CHOP21 [20]. However, the difference observed in this observational study may be down to the treating physician opting for the reduced treatment time provided by the biweekly regimen. Studies have demonstrated that CHOP14 and CHOP21 have equal efficacy and are associated with similar toxicities [20, 39].

This study had several limitations. As a result of the observational nature, clinic visits did not take place at fixed time points for all study participants, and selection bias could not be fully excluded. Only patients treated with R-CHOP were included, and there was no comparison to either untreated patients or patients treated with other chemotherapy regimens. In addition, the analyses of HRQoL by baseline characteristics, and associations between HRQoL and treatment response, were conducted retrospectively. Finally, although the EORTC QLQ-C30 is widely used in clinical trials to assess HRQoL, this questionnaire is not validated for lymphoma and so far, has not been widely used in daily clinical practice.

## Conclusions

These findings from the REFLECT study demonstrate the HRQoL benefit for treatment-naïve patients with DLBCL receiving SDZ-RTX-CHOP in a real-world setting. This is the first clinical or real-world study to demonstrate HRQoL benefits with a rituximab biosimilar in treatment-naïve patients with DLBCL. Patients experienced improvement from baseline to follow-up month 12 in emotional, physical, role, and social functioning. Symptom scales also demonstrated an improvement in pain, appetite loss, constipation, diarrhea, insomnia, fatigue, and nausea and vomiting. These results suggest that SDZ-RTX is an effective standard of care treatment that does not lead to impaired HRQoL in patients with DLBCL treated under real-world conditions.

### Electronic supplementary material

Below is the link to the electronic supplementary material.

Supplementary Material 1

## Data Availability

The data that support the findings of this study are not openly available due to reasons of sensitivity. They may be made available upon reasonable request to the corresponding author.
