# Supplementary material for: Quality of life assessment in diffuse large B-cell lymphoma (DLBCL) in REFLECT: a prospective, non-interventional, multicenter, German study, assessing Sandoz rituximab in combination with CHOP
Source: Ann Hematol. 2024 Jun 20;103(8):3165–78. doi: 10.1007/s00277-024-05850-5 (PMC11283426; doi:10.1007/s00277-024-05850-5)
Supplement: Supplementary file 1 — Supplementary Material 1 [file 277_2024_5850_MOESM1_ESM.pdf]

Supplementary Tables

Manuscript title: Quality of life assessment in diffuse large B-cell lymphoma (DLBCL) in REFLECT: a prospective, non-interventional, multicenter, German study, assessing Sandoz rituximab in combination with CHOP

Journal name: Annals of Hematology

Authors: Boris Kubuschok[1], Burkhard Otremba[2], Manfred Welslau[3], Julian Topaly[4], Thomas Wolff[5], Georg Lenz[6], Michael Grau[6], Larissa Bittencourt da Silva[7], Ines Brückmann[7], Tobias Foierl[7]

Author affiliations:

1. Universitätsklinikum Augsburg, Augsburg, Germany
2. Onkologische Praxis Oldenburg, Oldenburg, Germany
3. Onkologie Aschaffenburg, Aschaffenburg, Germany
4. MVZ Klinikum Osnabrück GmbH, Osnabrück, Germany
5. OncoResearch Lerchenfeld, Hamburg, Germany
6. Universitätsklinikum Münster, Department of Medicine A for Hematology, Oncology and Pneumology, Münster, Germany
7. Sandoz, Holzkirchen, Germany

Corresponding author: Boris Kubuschok (Boris.Kubuschok@uk-augsburg.de)

Supplementary Table 1 EORTC QLQ-C30 functional subscales by domain and visit (FAS population)

**Mean (SD)**	**Baseline**	**Mid-treatment**	**End of treatment**	**Follow-up**	
		**Month 3**	**Month 6**	**Month 9**	**Month 12**
**Global health status**	54.8 (24.0)	54.7 (20.8)	61.4 (18.9)	64.9 (18.6)	68.8 (21.3)
n	165	160	98	95	82
**Cognitive functioning**	82.9 (21.1)	76.7 (25.6)	80.3 (21.8)	78.2 (25.3)	80.9 (22.1)
n	165	160	98	95	82
**Emotional functioning**	63.2 (25.2)	67.4 (26.2)	74.0 (23.6)	71.8 (24.6)	75.1 (23.1)
n	165	160	98	95	82
**Physical functioning**	73.4 (25.4)	64.3 (24.1)	72.3 (21.8)	76.9 (20.8)	78.7 (22.1)
n	165	160	98	95	82
**Role functioning**	62.5 (34.5)	54.3 (31.6)	63.2 (28.6)	66.0 (30.0)	71.3 (31.9)
n	164	160	97	95	82
**Social functioning**	68.5 (31.5)	64.4 (30.9)	76.0 (23.2)	75.4 (25.0)	80.1 (24.9)
n	164	160	98	94	82

EORTC QLQ-C30, European Organization for Research and Treatment of Cancer quality of life questionnaire; FAS, full analysis set; SD, standard deviation.

Supplementary Table 2 EORTC QLQ-C30 symptom scales by domain and visit (FAS population)

Mean (SD)	Baseline	Mid-treatment	End of treatment	Follow-up	
		Month 3	Month 6	Month 9	Month 12
**Appetite loss**	28.7 (35.9)	27.5 (32.8)	15.0 (25.4)	16.8 (26.6)	11.1 (23.6)
n	165	159	98	95	81
**Constipation**	20.0 (31.2)	20.6 (31.7)	11.7 (22.1)	10.5 (22.9)	9.8 (19.9)
n	165	160	97	95	82
**Diarrhea**	11.5 (21.7)	13.0 (24.9)	9.2 (19.0)	7.4 (18.9)	7.3 (15.7)
n	165	159	98	95	82
**Dyspnea**	23.4 (31.9)	30.0 (31.4)	24.7 (28.6)	21.8 (26.1)	23.2 (27.6)
n	165	159	97	95	82
**Fatigue**	44.4 (28.3)	50.9 (26.5)	39.5 (23.1)	37.9 (25.1)	33.3 (25.8)
n	165	160	98	95	82
**Financial difficulties**	11.9 (24.4)	22.2 (29.9)	17.9 (30.8)	19.4 (31.6)	15.6 (28.9)
n	165	159	97	93	81
**Insomnia**	41.3 (35.6)	43.1 (33.6)	36.1 (31.3)	31.2 (32.9)	36.6 (33.0)
n	164	160	98	95	82
**Nausea and vomiting**	8.6 (18.7)	10.4 (17.8)	5.6 (13.3)	4.7 (13.0)	3.3 (10.3)
n	165	160	98	95	82
**Pain**	30.8 (33.3)	27.0 (29.6)	22.1 (27.7)	22.8 (27.9)	22.0 (27.8)
n	165	160	98	95	82

EORTC QLQ-C30, European Organization for Research and Treatment of Cancer quality of life questionnaire; FAS, full analysis set; SD, standard deviation.

Supplementary Table 3 Mean change from baseline to months 3, 6, 9, and 12 in EORTC QLQ-C30 domains stratified by age at baseline

		Mean change from baseline														
**Timepoint**	**Age (years)**	**Global health status**	**Cognitive functioning**	**Emotional functioning**	**Physical functioning**	**Role functioning**	**Social functioning**	**Appetite loss**	**Consti-pation**	**Diarrhea**	**Dyspnea**	**Fatigue**	**Financial difficulties**	**Insomnia**	**Nausea and vomiting**	**Pain**
**Mid treatment (month 3)**	**<65**	–0.69	–9.72	5.09	–10.86	–14.44	–6.67	–1.69	4.44	0.00	11.11	8.43	10.56	0.56	2.50	–3.61
	n	60	60	60	60	60	60	59	60	60	60	60	60	59	60	60
	**≥65**	–2.19	–7.68	–0.48	–12.62	–8.55	–8.22	5.26	2.63	2.22	4.89	7.97	10.67	4.82	1.97	–1.10
	n	76	76	76	76	76	75	76	76	75	75	76	75	76	76	76
	P-value	0.7609	0.6437	0.2225	0.6439	0.3286	0.7916	0.3412	0.7735	0.6157	0.2898	0.9272	0.9819	0.5114	0.8992	0.7081
**End of treatment (month 6)**	**<65**	2.96	–8.15	7.22	–5.93	–7.95	2.96	–6.67	0.74	–0.74	6.67	3.46	8.15	–1.52	–2.22	–5.56
	n	45	45	45	45	44	45	45	45	45	45	45	45	44	45	45
	**≥65**	4.89	–5.43	6.52	–8.30	–2.17	5.07	–6.52	–5.07	–3.62	–0.72	–1.69	3.62	–5.80	0.00	–2.90
	n	46	46	46	46	46	46	46	46	46	46	46	46	46	46	46
	P-value	0.7400	0.5394	0.9061	0.5897	0.4620	0.7617	0.9856	0.2989	0.5371	0.2302	0.3737	0.4661	0.5677	0.5871	0.7411
**Follow up month 9**	**<65**	0.39	–11.63	0.90	–0.15	–4.26	3.49	–6.98	–4.65	–3.88	0.00	–1.03	11.90	–6.35	–2.33	–1.94
	n	43	43	43	43	43	43	43	43	43	43	43	42	42	43	43
	**≥65**	12.06	–4.26	9.99	–4.72	–0.71	3.55	–5.67	–6.38	–4.26	0.71	1.06	3.55	–7.80	–2.48	–6.03
	n	47	47	47	47	47	47	47	47	47	47	47	47	47	47	47
	P-value	**0.0413**	0.2132	0.1448	0.2903	0.6838	0.9933	0.8742	0.7855	0.9440	0.9006	0.7232	0.1944	0.8610	0.9612	0.6224
**Follow up month 12**	**<65**	8.13	–7.50	10.83	1.17	2.50	8.75	–13.33	–13.33	–2.50	3.33	–6.39	5.83	–5.83	–3.75	–7.50
	n	40	40	40	40	40	40	40	40	40	40	40	40	40	40	40
	**≥65**	12.50	–2.63	4.39	–3.60	3.51	5.26	–11.40	–7.90	–4.39	6.14	–0.88	3.51	–8.77	–8.33	–1.75
	n	38	38	38	38	38	38	38	38	38	38	38	38	38	38	38
	P-value	0.5164	0.3662	0.3024	0.3463	0.9075	0.6445	0.8227	0.4437	0.6974	0.6457	0.3775	0.7010	0.7290	0.2944	0.4967

***P-values* in bold are < 0.05 (t-test)**

For global health status and functional scales, positive change from baseline = improvement in health status / functioning from baseline to timepoint; negative change from baseline = worsening in health status / functioning from baseline to timepoint.

For symptom scales, positive change from baseline = worsening in symptom from baseline to timepoint; negative change from baseline = improvement in symptom from baseline to timepoint.

EORTC QLQ-C30, European Organization for Research and Treatment of Cancer Core Quality of Life questionnaire.

At baseline: n=64 <65 years (except for insomnia, n=63); n=101 ≥65 years (except for role and social functioning, n=100).

Supplementary Table 4 Mean change from baseline to months 3, 6, 9, and 12 in EORTC QLQ-C30 domains stratified by sex

		Mean change from baseline														
**Timepoint**	**Sex**	**Global health status**	**Cognitive functioning**	**Emotional functioning**	**Physical functioning**	**Role functioning**	**Social functioning**	**Appetite loss**	**Consti-pation**	**Diarrhea**	**Dyspnea**	**Fatigue**	**Financial difficulties**	**Insomnia**	**Nausea and vomiting**	**Pain**
**Mid treatment (month 3)**	**Female**	–2.63	–11.87	–0.65	–13.66	–13.93	–10.88	3.24	3.65	1.85	8.80	9.51	11.11	3.70	4.57	–0.46
	n	73	73	73	73	73	72	72	73	72	72	73	72	72	73	73
	**Male**	–0.26	–4.76	5.03	–9.74	–7.94	–3.70	1.06	3.17	0.53	6.35	6.61	10.05	2.12	–0.53	–4.23
	n	63	63	63	63	63	63	63	63	63	63	63	63	63	63	63
	P-value	0.6301	0.1051	0.1996	0.2984	0.3336	0.2199	0.7644	0.9421	0.7711	0.6764	0.5615	0.8226	0.8057	0.2266	0.5723
**End of treatment (month 6)**	**Female**	–0.34	–12.58	0.00	–12.14	–15.65	–1.02	–5.44	–0.68	1.36	8.84	7.48	6.80	2.08	0.00	1.36
	n	49	49	49	49	49	49	49	49	49	49	49	49	48	49	49
	**Male**	8.93	0.00	14.88	–1.27	7.72	9.92	–7.94	–3.97	–6.35	–3.97	–6.88	4.76	–10.32	–2.38	–10.71
	n	42	42	42	42	41	42	42	42	42	42	42	42	42	42	42
	P-value	0.1091	**0.0029**	**0.0110**	**0.0122**	**0.0024**	0.1142	0.7563	0.5606	0.0975	**0.0365**	**0.0120**	0.7419	0.0984	0.5392	0.1321
**Follow up month 9**	**Female**	6.17	–12.67	5.33	–4.13	–2.00	3.33	–13.33	0.00	–2.00	–2.00	0.33	11.56	–4.08	–3.00	–1.67
	n	50	50	50	50	50	50	50	50	50	50	50	49	49	50	50
	**Male**	6.88	–1.67	6.04	–0.54	–2.92	3.75	2.50	–12.50	–6.67	3.33	–0.28	2.50	–10.83	–1.67	–7.08
	n	40	40	40	40	40	40	40	40	40	40	40	40	40	40	40
	P-value	0.9030	0.0520	0.9064	0.4148	0.9168	0.9523	**0.0400**	0.0528	0.3894	0.3488	0.9183	0.1521	0.4168	0.6605	0.5163
**Follow up month 12**	**Female**	6.82	–10.61	2.53	–4.62	–1.52	3.03	–16.67	–12.12	–3.03	6.82	0.51	6.82	–5.30	–7.96	–1.52
	n	44	44	44	44	44	44	44	44	44	44	44	44	44	44	44
	**Male**	14.71	1.96	14.38	3.33	8.82	12.26	–6.86	–8.82	–3.92	1.96	–9.15	1.96	–9.8	–3.43	–8.82
	n	34	34	34	34	34	34	34	34	34	34	34	34	34	34	34
	P-value	0.2444	**0.0188**	**0.0438**	0.1168	0.2354	0.2240	0.2182	0.6453	0.8554	0.4047	0.1231	0.4294	0.5984	0.2698	0.3843

***P-values* in bold are < 0.05 (t-test)**

For global health status and functional scales, positive change from baseline = improvement in health status / functioning from baseline to timepoint; negative change from baseline = worsening in health status / functioning from baseline to timepoint.

For symptom scales, positive change from baseline = worsening in symptom from baseline to timepoint; negative change from baseline = improvement in symptom from baseline to timepoint.

EORTC QLQ-C30, European Organization for Research and Treatment of Cancer Core Quality of Life questionnaire.

At baseline: n=86 females (except for insomnia, role functioning and social functioning, n=85); n=79 males.

Supplementary Table 5 Mean change from baseline to months 3, 6, 9, and 12 in EORTC QLQ-C30 domains stratified by disease stage at baseline

		**Mean change from baseline**														
**Timepoint**	**Ann Arbor disease stage**	**Global health status**	**Cognitive functioning**	**Emotional functioning**	**Physical functioning**	**Role functioning**	**Social functioning**	**Appetite loss**	**Consti-pation**	**Diarrhea**	**Dyspnea**	**Fatigue**	**Financial difficulties**	**Insomnia**	**Nausea and vomiting**	**Pain**
**Mid treatment (month 3)**	**I/II**	–6.25	–10.97	0.88	–12.74	–15.57	–8.11	6.67	7.02	2.19	7.46	12.13	10.67	1.78	2.41	–0.44
	n	76	76	76	76	76	76	75	76	76	76	76	75	75	76	76
	**III/IV**	4.52	–5.65	3.44	–10.21	–3.95	–5.17	–5.08	–1.13	0.00	8.05	2.26	10.17	2.82	1.98	–5.37
	n	59	59	59	59	59	58	59	59	58	58	59	59	59	59	59
	P-value	**0.0287**	0.2498	0.5789	0.5053	0.0569	0.6107	0.1019	0.2210	0.6344	0.9210	**0.0471**	0.9170	0.8693	0.9205	0.4636
**End of treatment (month 6)**	**I/II**	–1.54	–8.64	4.22	–9.38	-9.88	0.93	–3.70	–1.23	0.00	4.32	4.12	6.17	–6.29	1.23	0.62
	n	54	54	54	54	54	54	54	54	54	54	54	54	53	54	54
	**III/IV**	11.94	–4.05	10.74	–3.83	2.32	8.56	–10.81	–3.60	–5.41	0.90	–3.90	5.41	0.00	–4.50	–11.26
	n	37	37	37	37	36	37	37	37	37	37	37	37	37	37	37
	P-value	**0.0207**	0.3361	0.2795	0.2132	0.1265	0.2794	0.3827	0.6796	0.2542	0.6194	0.1720	0.9029	0.4112	0.2170	0.1445
**Follow up month 9**	**I/II**	3.37	–9.29	4.43	–3.20	–5.45	3.21	–0.64	–1.92	–1.92	–0.64	0.85	11.11	–11.76	–1.60	0.64
	n	52	52	52	52	52	52	52	52	52	52	52	51	51	52	52
	**III/IV**	10.74	–5.70	7.31	–1.62	1.75	3.95	–14.04	–10.53	–7.02	1.75	–1.02	2.63	–0.88	–3.51	–10.53
	n	38	38	38	38	38	38	38	38	38	38	38	38	38	38	38
	P-value	0.2053	0.5725	0.6499	0.7358	0.4137	0.9156	0.1295	0.1878	0.3501	0.6764	0.7537	0.1832	0.1915	0.5946	0.1818
**Follow up month 12**	**I/II**	7.69	–5.77	7.59	–4.74	–4.17	3.21	–5.13	–8.97	–3.21	5.77	–0.43	8.33	–7.69	–1.92	–1.92
	n	52	52	52	52	52	52	52	52	52	52	52	52	52	52	52
	**III/IV**	15.38	–3.85	7.91	6.03	17.31	14.74	–26.92	–14.10	–3.85	2.56	–10.26	–2.56	–6.41	–14.10	–10.26
	n	26	26	26	26	26	26	26	26	26	26	26	26	26	26	26
	P-value	0.2808	0.7369	0.9613	**0.0424**	**0.0175**	0.1474	**0.0335**	0.5879	0.9008	0.6206	0.1359	0.0895	0.8867	**0.0214**	0.3454

***P-values* in bold are < 0.05 (t-test)**

For global health status and functional scales, positive change from baseline = improvement in health status / functioning from baseline to timepoint; negative change from baseline = worsening in health status / functioning from baseline to timepoint.

For symptom scales, positive change from baseline = worsening in symptom from baseline to timepoint; negative change from baseline = improvement in symptom from baseline to timepoint.

EORTC QLQ-C30, European Organization for Research and Treatment of Cancer Core Quality of Life questionnaire.

At baseline: n=90 Stage I/II (except for insomnia, n=89); n=74 Stage III/IV (except for role and social functioning, n=73).

Supplementary Table 6 Mean change from baseline to months 3, 6, 9, and 12 in EORTC QLQ-C30 domains stratified by IPI score at baseline

		**Mean change from baseline**														
**Timepoint**	**IPI score**	**Global health status**	**Cognitive functioning**	**Emotional functioning**	**Physical functioning**	**Role functioning**	**Social functioning**	**Appetite loss**	**Consti-pation**	**Diarrhea**	**Dyspnea**	**Fatigue**	**Financial difficulties**	**Insomnia**	**Nausea and vomiting**	**Pain**
**Mid treatment (month 3)**	**0–2**	–7.98	–16.20	–1.49	–16.01	–18.54	–9.76	9.05	7.51	4.69	10.80	14.79	13.81	3.81	6.10	4.23
	n	71	71	71	71	71	70	70	71	71	71	71	70	70	71	71
	**3–5**	6.10	0.00	5.42	–6.72	2.03	–2.44	–13.01	–4.07	1.67	1.67	–2.30	6.50	–4.07	–5.28	–9.35
	n	41	41	41	41	41	41	41	41	40	40	41	41	41	41	41
	P-value	**0.0106**	**0.0018**	0.1703	**0.0376**	**0.0030**	0.2740	**0.0061**	0.1204	0.5576	0.1992	**0.0032**	0.1889	0.2906	**0.0309**	0.0835
**End of treatment (month 6)**	**0–2**	1.57	–9.43	6.29	–9.56	–11.54	0.94	–4.40	–1.89	1.26	5.03	6.29	9.43	–1.28	0.31	2.52
	n	53	53	53	53	52	53	53	53	53	53	53	53	52	53	53
	**3–5**	11.11	1.39	11.00	–0.90	11.11	11.81	–18.06	–4.17	–8.33	–6.94	–12.5	–1.39	–13.89	–6.94	–20.83
	n	24	24	24	24	24	24	24	24	24	24	24	24	24	24	24
	P-value	0.1889	0.0548	0.4865	0.1050	**0.0156**	0.1878	0.1454	0.7782	0.0525	0.1089	**0.0074**	0.1231	0.1587	0.2541	**0.0103**
**Follow up month 9**	**0–2**	3.27	–12.75	0.87	–6.40	–11.76	0.33	0.65	–1.31	0.65	1.96	5.34	11.33	–4.67	–1.31	3.92
	n	51	51	51	51	51	51	51	51	51	51	51	50	50	51	51
	**3–5**	12.82	–0.64	11.75	2.24	12.18	8.33	–19.23	–15.38	–7.69	0.00	–7.69	1.28	–8.97	–5.77	–17.95
	n	26	26	26	26	26	26	26	26	26	26	26	26	26	26	26
	P-value	0.1600	0.0758	0.1260	0.0947	**0.0161**	0.3118	**0.0401**	0.1457	0.1569	0.7720	0.0592	0.1638	0.6387	0.3126	**0.0167**
**Follow up month 12**	**0–2**	8.69	–6.03	4.67	–3.69	–3.55	3.19	–7.80	–7.09	–2.13	5.67	–0.95	6.38	–3.55	–5.32	–1.06
	n	47	47	47	47	47	47	47	47	47	47	47	47	47	47	47
	**3–5**	16.18	–3.92	14.05	5.49	25.49	20.59	–35.29	–19.61	–9.80	3.92	–15.03	3.92	–21.57	–17.65	–15.69
	n	17	17	17	17	17	17	17	17	17	17	17	17	17	17	17
	P-value	0.3701	0.7511	0.2239	0.1550	**0.0070**	0.0779	**0.0104**	0.3061	0.3068	0.8189	0.0766	0.7297	0.0737	0.0623	0.2855

***P-values* in bold are < 0.05 (t-test)**

For global health status and functional scales, positive change from baseline = improvement in health status / functioning from baseline to timepoint; negative change from baseline = worsening in health status / functioning from baseline to timepoint.

For symptom scales, positive change from baseline = worsening in symptom from baseline to timepoint; negative change from baseline = improvement in symptom from baseline to timepoint.

EORTC QLQ-C30, European Organization for Research and Treatment of Cancer Core Quality of Life questionnaire; IPI, International Prognostic Index.

At baseline: n=83 IPI 0–2 (except for social functioning and insomnia, n=82); n=56 IPI 3–4 (except for role functioning, n=55).

Supplementary Table 7 Mean change from baseline to months 3, 6, 9, and 12 in EORTC QLQ-C30 domains stratified by use of key concomitant medications at baseline

		Mean change from baseline														
**Timepoint**	**Use of key CM**	**Global health status**	**Cognitive functioning**	**Emotional functioning**	**Physical functioning**	**Role functioning**	**Social functioning**	**Appetite loss**	**Consti-pation**	**Diarrhea**	**Dyspnea**	**Fatigue**	**Financial difficulties**	**Insomnia**	**Nausea and vomiting**	**Pain**
**Mid treatment (month 3)**	**No**	–0.93	–6.48	3.70	–9.63	–4.63	–0.93	–1.96	–18.52	–1.85	0.00	4.32	–1.85	14.82	0.00	–8.33
	n	18	18	18	18	18	18	17	18	18	18	18	18	18	18	18
	**Yes**	–1.62	–8.90	1.72	–12.18	–12.15	–8.55	2.83	6.78	1.71	8.83	8.76	12.54	1.14	2.54	–1.27
	n	118	118	118	118	118	117	118	118	117	117	118	117	117	118	118
	P-value	0.9229	0.7094	0.7672	0.5295	0.4099	0.3752	0.6619	**0.0082**	0.5934	0.3040	0.5460	**0.0363**	0.1472	0.7584	0.4728
**End of treatment (month 6)**	**No**	–20.24	11.90	1.19	–12.38	–11.90	4.76	4.76	–4.76	–9.52	–4.76	9.52	–14.29	9.52	9.52	26.19
	n	7	7	7	7	7	7	7	7	7	7	7	7	7	7	7
	**Yes**	5.95	–8.33	7.34	–6.69	–4.42	3.97	–7.54	–1.98	–1.59	3.57	0.13	7.54	–4.82	–1.98	–6.75
	n	84	84	84	84	83	84	84	84	84	84	84	84	83	84	84
	P-value	**0.0146**	**0.0132**	0.5808	0.4900	0.6101	0.9515	0.4126	0.7929	0.3645	0.4720	0.3871	0.0572	0.3072	0.1318	**0.027**
**Follow up month 9**	**No**	–3.47	2.78	13.89	–1.67	–5.55	15.28	–19.45	–16.67	0.00	–11.11	–6.02	–11.11	–16.67	1.39	4.17
	n	12	12	12	12	12	12	12	12	12	12	12	12	12	12	12
	**Yes**	8.01	–9.40	4.38	–2.67	–1.92	1.71	–4.27	–3.85	–4.70	2.14	1.00	10.39	–5.63	–2.99	–5.34
	n	78	78	78	78	78	78	78	78	78	78	78	77	77	78	78
	P-value	0.1745	0.1613	0.3004	0.8761	0.8345	0.1800	0.2076	0.1766	0.5534	0.2964	0.4192	**0.0185**	0.3616	0.3527	0.4354
**Follow up month 12**	**No**	23.33	3.33	13.33	6.67	13.33	31.67	–30.00	–43.33	–13.33	0.00	–14.44	–3.33	–23.33	–13.33	–3.33
	n	10	10	10	10	10	10	10	10	10	10	10	10	10	10	10
	**Yes**	8.33	–6.37	6.86	–2.30	1.47	3.43	–9.8	–5.88	–1.96	5.39	–2.12	5.88	–4.90	–4.90	–4.90
	n	68	68	68	68	68	68	68	68	68	68	68	68	68	68	68
	P-value	0.1344	0.2273	0.4854	0.2347	0.3596	**0.0107**	0.1138	**0.0425**	0.1140	0.7425	0.1857	0.3114	0.1433	0.1962	0.9001

***P-values* in bold are < 0.05 (t-test)**

For global health status and functional scales, positive change from baseline = improvement in health status / functioning from baseline to timepoint; negative change from baseline = worsening in health status / functioning from baseline to timepoint.

For symptom scales, positive change from baseline = worsening in symptom from baseline to timepoint; negative change from baseline = improvement in symptom from baseline to timepoint.

CM, concomitant medication.

Key CM defined as corticosteroids for systemic use, analgesics, antiemetics and antinauseants, antibacterial for systemic use, and antihistamines for systemic use.

At baseline: n=23 for no use of key CM; n=142 for use of key CM (except for insomnia, role functioning and social functioning, n=141).

Supplementary Table 8 Mean change from baseline to months 3, 6, 9, and 12 in EORTC QLQ-C30 domains stratified by presence of any medical event in patient history at baseline

		Mean change from baseline														
**Timepoint**	**Medical event in history**	**Global health status**	**Cognitive functioning**	**Emotional functioning**	**Physical functioning**	**Role functioning**	**Social functioning**	**Appetite loss**	**Consti-pation**	**Diarrhea**	**Dyspnea**	**Fatigue**	**Financial difficulties**	**Insomnia**	**Nausea and vomiting**	**Pain**
**Mid treatment (month 3)**	**No**	–0.21	–7.27	–2.07	–9.12	–16.24	–8.55	0.00	2.56	–1.75	4.39	11.97	14.53	7.02	0.00	–2.14
	n	39	39	39	39	39	39	39	39	38	38	39	39	38	39	39
	**Yes**	–2.06	–9.11	3.61	–12.94	–9.11	–7.12	3.13	3.78	2.41	8.93	6.64	9.03	1.37	3.09	–2.23
	n	97	97	97	97	97	96	96	97	97	97	97	96	97	97	97
	P-value	0.7325	0.7048	0.1904	0.3587	0.2963	0.8248	0.6965	0.8257	0.4088	0.4841	0.3332	0.2887	0.4301	0.5146	0.9895
**End of treatment (month 6)**	**No**	4.72	–7.78	2.78	–4.00	–8.05	–3.33	–6.67	–4.44	1.11	0.00	2.96	14.44	–8.05	0.00	–6.67
	n	30	30	30	30	29	30	30	30	30	30	30	30	29	30	30
	**Yes**	3.55	–6.28	8.88	–8.66	–3.55	7.65	–6.56	–1.09	–3.83	4.37	–0.18	1.64	–1.64	–1.64	–3.00
	n	61	61	61	61	61	61	61	61	61	61	61	61	61	61	61
	P-value	0.8497	0.7508	0.3330	0.3181	0.5932	0.1352	0.9897	0.5759	0.3194	0.5057	0.6099	**0.0489**	0.4266	0.7066	0.6159
**Follow up month 9**	**No**	7.22	–5.00	4.44	2.00	2.78	3.89	–16.67	–6.67	–4.44	–4.44	–5.56	6.67	–12.64	–5.56	–9.44
	n	30	30	30	30	30	30	30	30	30	30	30	30	29	30	30
	**Yes**	6.11	–9.17	6.25	–4.81	–5.00	3.33	–1.11	–5.00	–3.89	2.78	2.87	7.91	–4.44	–0.83	–1.39
	n	60	60	60	60	60	60	60	60	60	60	60	59	60	60	60
	P-value	0.8561	0.5081	0.7856	0.1410	0.3995	0.9396	0.0718	0.7866	0.9227	0.2279	0.1771	0.8335	0.3525	0.1634	0.3592
**Follow up month 12**	**No**	12.78	–4.44	11.85	4.00	4.44	2.78	–17.78	–12.22	–7.78	–6.67	–10.00	5.56	–14.44	–6.11	–14.44
	n	30	30	30	30	30	30	30	30	30	30	30	30	30	30	30
	**Yes**	8.68	–5.56	5.09	–4.38	2.08	9.72	–9.03	–9.72	–0.69	11.80	0.23	4.17	–2.78	–5.90	1.39
	n	48	48	48	48	48	48	48	48	48	48	48	48	48	48	48
	P-value	0.5543	0.8414	0.2879	0.1050	0.7913	0.3703	0.3212	0.7322	0.1526	**0.0024**	0.1087	0.8249	0.1781	0.9631	0.0621

***P-values* in bold are < 0.05 (t-test)**

For global health status and functional scales, positive change from baseline = improvement in health status / functioning from baseline to timepoint; negative change from baseline = worsening in health status / functioning from baseline to timepoint.

For symptom scales, positive change from baseline = worsening in symptom from baseline to timepoint; negative change from baseline = improvement in symptom from baseline to timepoint.

At baseline: n=46 for no history of medical event (except for role functioning and insomnia, n=45); n=119 for history of medical event (except for social functioning, n=118).

Supplementary Table 9 Mean change from baseline to months 3, 6, 9, and 12 in EORTC QLQ-C30 domains stratified by presence of any serious medical event in patient history at baseline

		**Mean change from baseline**														
**Timepoint**	**Serious medical event in history**	**Global health status**	**Cognitive functioning**	**Emotional functioning**	**Physical functioning**	**Role functioning**	**Social functioning**	**Appetite loss**	**Consti-pation**	**Diarrhea**	**Dyspnea**	**Fatigue**	**Financial difficulties**	**Insomnia**	**Nausea and vomiting**	**Pain**
**Mid treatment (month 3)**	**No**	–1.22	–7.18	3.06	–11.10	–9.91	–8.05	0.87	2.59	–0.29	8.41	6.94	11.49	2.03	1.72	–2.44
	**n**	116	116	116	116	116	116	115	116	115	115	116	116	115	116	116
	**Yes**	–3.33	–16.67	–4.31	–16.17	–18.33	–4.39	10.00	8.33	10.00	3.33	15.28	5.26	8.33	5.00	–0.83
	**n**	20	20	20	20	20	19	20	20	20	20	20	19	20	20	20
	P-value	0.7598	0.1250	0.2495	0.3402	0.3345	0.6635	0.3712	0.5354	0.1053	0.5379	0.2350	0.1891	0.4863	0.5889	0.8643
**End of treatment (month 6)**	**No**	5.59	–6.12	6.33	–5.93	–3.42	2.53	–7.59	–1.69	–2.53	2.95	–0.42	8.02	–5.98	–1.27	–7.17
	**n**	79	79	79	79	78	79	79	79	79	79	79	79	78	79	79
	**Yes**	–6.95	–11.11	10.42	–15.00	–15.28	13.89	0.00	–5.56	0.00	2.78	9.26	–8.33	11.11	0.00	15.28
	**n**	12	12	12	12	12	12	12	12	12	12	12	12	12	12	12
	P-value	0.1418	0.4445	0.6413	0.1608	0.3041	0.2675	0.5209	0.6423	0.7139	0.9770	0.1029	0.0708	0.1210	0.8342	0.0564
**Follow up month 9**	**No**	6.39	–5.84	6.42	–1.15	–1.73	5.19	–8.23	–5.63	–4.76	0.87	–0.79	6.58	–7.89	–4.11	–6.06
	**n**	77	77	77	77	77	77	77	77	77	77	77	76	76	77	77
	**Yes**	7.05	–19.23	1.07	–10.77	–6.41	–6.41	5.13	–5.13	0.00	–2.56	5.13	12.82	–2.56	7.69	7.69
	**n**	13	13	13	13	13	13	13	13	13	13	13	13	13	13	13
	P-value	0.9355	0.1107	0.5475	0.1204	0.7059	0.2362	0.2517	0.9568	0.3349	0.5437	0.4810	0.4858	0.6494	**0.0084**	0.2423
**Follow up month 12**	**No**	11.92	–4.17	8.68	0.28	5.09	8.33	–14.35	–10.65	–4.17	4.63	–5.25	4.63	–7.87	–6.94	–7.18
	**n**	72	72	72	72	72	72	72	72	72	72	72	72	72	72	72
	**Yes**	–9.72	–16.67	–4.17	–18.33	–22.22	–8.34	11.11	–11.11	5.56	5.56	14.82	5.56	0.00	5.56	25.00
	**n**	6	6	6	6	6	6	6	6	6	6	6	6	6	6	6
	P-value	0.0844	0.2151	0.2684	**0.0476**	0.0905	0.2379	0.1120	0.9723	0.2836	0.9356	0.0846	0.9357	0.6206	0.1258	**0.0373**

***P-values* in bold are < 0.05 (t-test)**

For global health status and functional scales, positive change from baseline = improvement in health status / functioning from baseline to timepoint; negative change from baseline = worsening in health status / functioning from baseline to timepoint.

For symptom scales, positive change from baseline = worsening in symptom from baseline to timepoint; negative change from baseline = improvement in symptom from baseline to timepoint.

Serious medical event defined as any of the following: cardiac failure, left ventricular failure, renal failure, hepatic cirrhosis, asthma, chronic obstructive pulmonary disease, pulmonary embolism, pulmonary fibrosis, Parkinson's disease, autoimmune thyroiditis, rheumatoid arthritis, or psoriasis.

At baseline: n=140 for no history of serious medical event (except for role functioning and insomnia, n=139); n=25 for history of serious medical event (except for social functioning, n=24).

Supplementary Table 10 Mean baseline HRQoL by EORTC QLQ-C30 domain in patients reaching a CR or PR

Response		Global health status	Cognitive functioning	Emotional functioning	Physical functioning	Role functioning	Social functioning	Appetite loss	Constipation	Diarrhea	Dyspnea	Fatigue	Financial difficulties	Insomnia	Nausea and vomiting	Pain
**Best response during study**	**CR**	56.54	86.29	64.82	78.26	68.54	70.56	24.30	17.13	8.41	22.12	40.08	12.77	38.68	9.50	25.70
	n	107	107	107	107	107	107	107	107	107	107	107	107	106	107	107
	**PR**	52.89	78.23	60.26	64.90	51.39	62.15	38.10	28.57	17.01	24.49	51.81	11.56	44.22	7.82	40.48
	n	49	49	49	49	48	48	49	49	49	49	49	49	49	49	49
	P-value	0.3795	**0.0181**	0.2868	**0.0018**	**0.0038**	0.1210	**0.0259**	**0.0364**	**0.0412**	0.6559	**0.0153**	0.7795	0.3588	0.5760	**0.0098**
**Response at end of treatment**	**CR**	57.75	88.43	65.70	80.37	71.99	71.30	21.30	16.20	6.94	18.06	35.80	12.50	34.72	6.02	22.22
	n	72	72	72	72	72	72	72	72	72	72	72	72	72	72	72
	**PR**	53.78	80.00	62.96	69.96	56.76	64.86	34.22	25.33	13.78	26.67	49.26	13.33	45.50	11.11	36.22
	n	75	75	75	75	74	74	75	75	75	75	75	75	74	75	75
	P-value	0.3222	**0.0091**	0.4996	**0.0103**	**0.0072**	0.2109	**0.0289**	0.0844	**0.0347**	0.0898	**0.0033**	0.8433	0.0611	0.1090	**0.0105**

***P-values* in bold are < 0.05 (t-test)**

CR, complete response; EORTC QLQ-C30, European Organization for Research and Treatment of Cancer Core Quality of Life questionnaire; HRQoL, health-related quality of life.PR, partial response;
